# From “physical repair” to “mental resonance”: spatial perception and behavioral reshaping pathways in the renewal of lost under-bridge spaces

**DOI:** 10.3389/fpsyg.2026.1906471

**Published:** 2026-08-11

**Authors:** Yuelan Xu, Yujing Zhang, Xiaochun Zhan

**Affiliations:** School of Art and Media, Xianda College of Economics and Humanities Shanghai International Studies University, Shanghai, China

**Keywords:** behavioral reshaping, environmental renewal, mental perception, SEM model, under-bridge space

## Abstract

**Introduction:**

Urban under-bridge spaces are often regarded as typical “lost spaces” due to ambiguous boundaries, limited functions, and fragmented management. While existing regeneration practices have largely focused on functional supplementation, safety governance, and the maintenance of spatial order, the mechanisms through which physical environmental qualities are translated into public interaction behaviors via users’ psychological experiences remain theoretically underspecified.

**Methods:**

Grounded in environmental behavior theory and adopting a mixed-methods research design, this study takes the under-bridge space of the Lujiazui Waterfront Ring Bridge in Shanghai as the research object. Empirical data were collected through a pedestrian questionnaire survey, and a structural equation model (SEM) was constructed to operationalize the following theoretical pathway: Physical Environment - Mental Perception - Behavioral Reshaping.

**Results:**

The analysis of 357 valid questionnaires shows that FS, AC, and RC all exert significant effects on MP (*β* = 0.181, *p* = 0.006; *β* = 0.221, *p* < 0.001; *β* =  0.409, *p* < 0.001). FS and RC also have significant direct effects on BR (*β* = 0.205, *p* = 0.001; *β* = 0.280, *p* = 0.001), whereas the direct path from AC to BR is not supported (*β* = 0.116, *p* = 0.063). The Bootstrap mediation test with 5,000 resamples indicates that MP plays a partial mediating role between FS and BR, with an indirect effect whose 95% confidence interval is [0.016, 0.138], and between RC and BR, with a 95% confidence interval of [0.056, 0.240]. In contrast, MP fully mediates the relationship between AC and BR; the 95% confidence interval for the path from AC to BR was [−0.010, 0.288], and MP plays a full mediating role between AC and BR, with a confidence interval of [0.024, 0.151].The model demonstrates good fit indices (χ^2^/df = 1.237, RMSEA = 0.026, GFI = 0.950, CFI = 0.988).

**Discussion:**

These findings clarify the theoretically ambiguous relationship between spatial regeneration and behavioral outcomes by demonstrating that mental perception constitutes a crucial and differentially operative mediating pathway: while functional and reachability dimensions retain direct behavioral effects independent of perception, aesthetic and comfort qualities exert their influence exclusively through the psychological experience of users. Accordingly, under-bridge space regeneration should be seen as a continuous, integrative process involving physical environment upgrades, psychological experience enhancements, and social behavior generation. These findings offer empirical and theoretical insights for regenerating under-bridge spaces, reconstructing publicness, and enhancing urban space quality.

## 1 Introduction

As urbanization continues to advance, the construction of transport infrastructure has alleviated congestion and improved mobility efficiency, while also continuously reshaping the organization of urban space. In large and medium-sized cities characterized by high concentrations of population and industry, three-dimensional transport facilities such as viaducts divide urban space into fragments of varying scales. The spaces beneath them often feature ambiguous boundaries, complex structures, insufficient daylight, limited functions, and overlapping management responsibilities. As a result, they have long remained underused and socially neglected, and are commonly referred to as lost spaces or negative spaces in the city. In Shanghai, for example, the three-dimensional network of transport infrastructure between the Outer Ring and Inner Ring has fragmented urban areas, and the under-bridge spaces below this network have become residual urban fragments whose potential value is underestimated. In megacities with scarce land resources and high population density, however, under-bridge spaces are not merely appendages of transport infrastructure; they are stock spatial resources with potential public, cultural, and renewal value. In recent years, as the concept of urban renewal has shifted from large-scale demolition and reconstruction toward refined governance, stock optimization, and quality improvement, the reuse of under-bridge spaces has gradually become an important topic in urban spatial restructuring. For example, Shanghai launched an urban micro-renewal program from 2018 to 2019 focusing on the activation of under-bridge spaces; by selecting pilot bridges in different locations and of different types, the program explored feasible pathways for diversified reuse (Shanghai Urban Planning and Design Research Institute, 2023). In 2026, the Shanghai Municipal Bureau of Planning and Natural Resources issued formal documents on the renewal of spaces beneath urban viaducts, clarifying that such spaces may accommodate public parking lots, football fields, basketball courts, and other sports facilities (Shanghai Municipal Bureau of Planning and Natural Resources, 2026). These practices suggest that under-bridge spaces are gradually shifting from passive residual spaces to public spatial resources that can be reorganized and activated.

Nevertheless, existing under-bridge renewal practices still tend to be strongly function-oriented, emphasizing practicality, safety, and order while paying relatively limited attention to aesthetic expression, cultural identity, emotional experience, and public interaction. In other words, many renewal projects have completed environmental remediation at the physical level without necessarily rebuilding the interactive relationship between space and people. From the perspective of environment-behavior studies, the lost quality of under-bridge spaces is not merely a problem of inadequate facilities or environmental deterioration. More fundamentally, it derives from a mismatch between the material environment and human behavioral needs. When a space provides appropriate conditions for staying, a clear sense of territory, and positive perceptual stimuli, social behaviors such as staying, communicating, and participating are more likely to occur. Conversely, when a space conveys signals of oppression, alienation, or insecurity, people tend to pass through quickly, avoid it, or leave. On this basis, this study takes function and safety, aesthetics and comfort, and reachability and catalysis as the core dimensions of the physical environment of under-bridge spaces; takes mental perception as the psychological pathway connecting spatial environment and behavioral change; and takes behavioral reshaping as the outcome variable of spatial activation. It thereby investigates how under-bridge spaces can move beyond physical repair toward mental resonance.

## 2 Theoretical basis and research hypotheses

**Figure 1:**
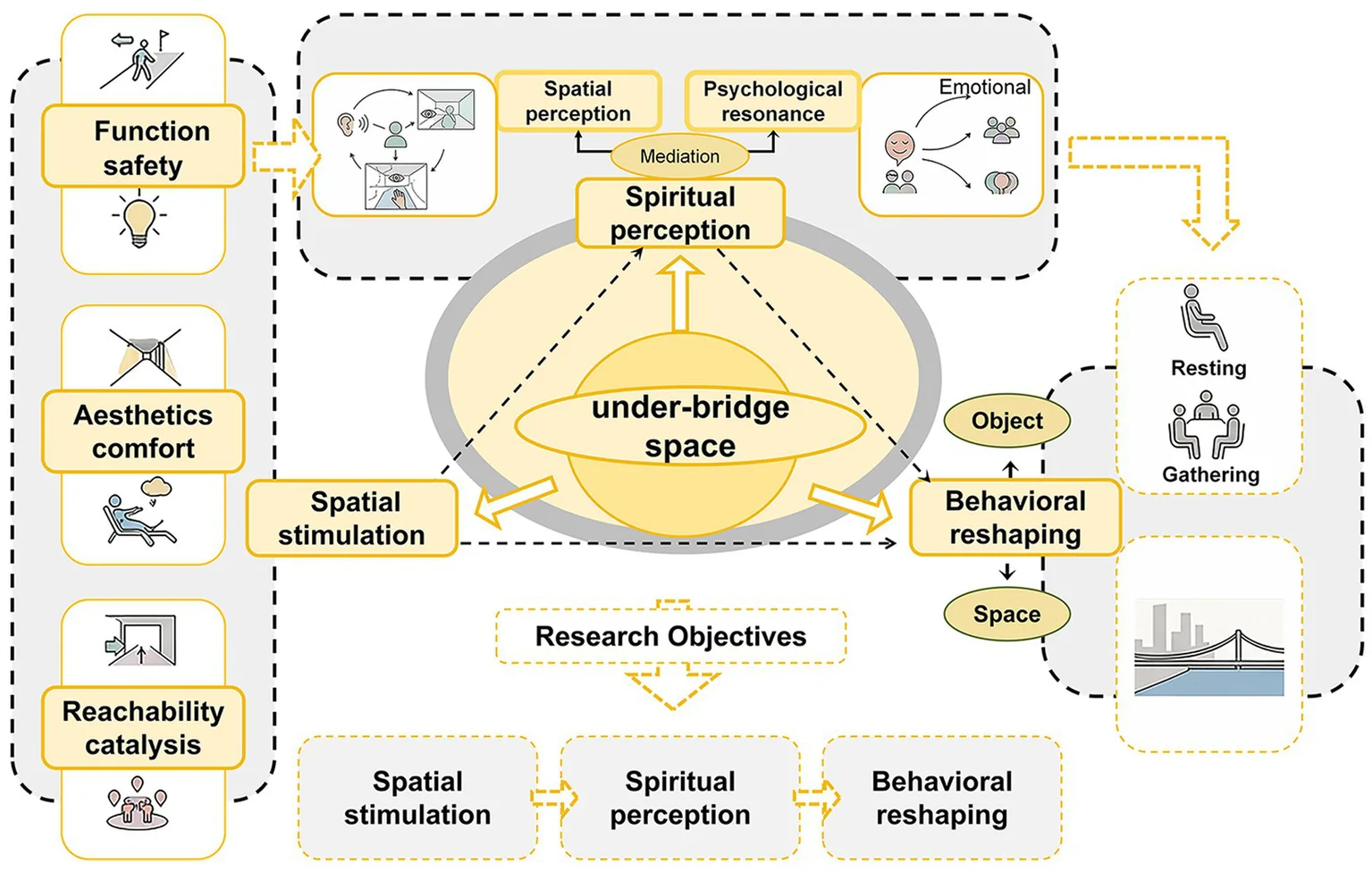
Spatial stimulation—spatial perception—behavioral reshaping relationship diagram.

Müller et al. argue that spatial intervention models, such as physical restorative environments, salutogenesis, and biopsychosocial health, can transform urban residual spaces from sources of stress into mental health resources through small-scale, low-threshold, and resource-efficient urban design interventions, thereby promoting the occurrence of behaviors (Müller et al., 2023). This study aims to examine how the reconstruction of the physical reconstruction of under-bridge spaces stimulates users’ mental perception and further promotes the reshaping of their everyday behavior. To this end, this chapter examines the relationships among physical space, psychological perception, and social behavior from an environmental-behavioral perspective, develops a theoretical framework, and proposes the corresponding research hypotheses (Figure 1).

### 2.1 Dialectical reflections on environment-behavior studies and the production of space

Environment-behavior studies are often regarded as closely related to, or overlapping with, environmental psychology. This field mainly examines the interactions and regularities among human behavior, psychological processes, and the physical environment, including both natural and built environments. It integrates knowledge from architecture, urban planning, psychology, sociology, anthropology, and other disciplines. As an important tool for understanding and analyzing spatial phenomena, environment-behavior studies provide a critical theoretical perspective for the reconstruction of under-bridge spaces and the reshaping of user behavior.

Environment-behavior theory primarily investigates behavioral regularities in specific environments and how environmental factors influence human psychology and behavior (Bechtel and Churchman, 2002). Here, environment refers not only to natural settings but also to built spaces such as architectural interiors, urban streets, and public open spaces. Behavior likewise refers not only to visible bodily activity, but also to perception, cognition, emotional experience, spatial choice, social interaction, sense of belonging, sense of safety, and satisfaction (Nasar, 2007). In this sense, environment-behavior theory is a theoretical system concerning the interaction between people and environments. It seeks to explain how environments influence behavior, how people perceive, use, evaluate, and transform environments, and how architecture, urban planning, landscape design, and public policy can be grounded in human-centered evidence.

The theory of the production of space, represented by scholars such as Henri Lefebvre and David Harvey, profoundly reveals that space is neither natural nor given, but rather a product jointly produced by social relations, power struggles, ideology, and everyday practices (Bao, 2002). In The Production of Space, Lefebvre argues that space is not a single physical entity. He decomposes spatial production into three interrelated dimensions: representations of space, spatial practice, and representational spaces. These three dimensions are not independent stages or levels, but mutually related and inseparable logical dimensions that form a dialectical social product (Lefebvre, 1974). This theory highlights the complexity and dynamic evolution of space across material, cognitive, and social dimensions. As a powerful tool for understanding and analyzing spatial phenomena, it provides a deep dialectical perspective for the reconstruction of under-bridge art spaces and the reshaping of user behavior. In the context of urban under-bridge spaces, the original spatial practice is mainly the shaded zone beneath transport infrastructure, characterized by limited functions, negative environmental qualities, and low utilization. With the rise of urban renewal and public art interventions, however, under-bridge spaces are being reexamined and activated. Environmental transformation strategies such as spatial remodeling, cultural communication, and artistic intervention seek to break their inherent negative attributes and convert them into social spaces with public value. From the perspective of the production of space, renewed under-bridge spaces can produce new social practices and meanings, thereby reshaping patterns of gathering, communication, and creative behavior (Song et al., 2025).

### 2.2 Relationships between physical environmental elements and mental perception

Function and safety (FS) are widely regarded as key factors influencing users’ mental perception (Lak et al., 2019). For under-bridge spaces, high-quality function means that the space can respond to the practical needs of pedestrians, community residents, and leisure users, for example by providing sheltered resting places, recreational and sports facilities, and necessary service amenities. Safety means that the space offers clear sightlines, stable lighting, and controllable traffic disturbance. In urban public spaces in particular, safety and practicality form the basis on which citizens choose and use a space (Cerin et al., 2006). When the functional design of an under-bridge space meets users’ expectations and its safety attributes are sufficiently secured, individuals’ trust, comfort, and identification with the space increase accordingly. Therefore, sound functional provision and spatial safety are essential for establishing and maintaining positive mental perception of under-bridge spaces.

*H1*: Function and safety have a significant positive effect on users’ mental perception.

Aesthetics and comfort (AC) constitute another important factor influencing users’ mental perception. This dimension covers the visual attractiveness of under-bridge spaces in terms of landscape greening, art installations, cultural communication, and related design features (Jiang et al., 2025), as well as the pleasure generated by the experience of use. Previous studies have examined the role of aesthetics and comfort in public spaces in enhancing users’ mental perception. A high level of aesthetics and comfort can strengthen individuals’ experience of, and identification with, under-bridge spaces (Fedorczak-Cisak et al., 2022). Moreover, studies have shown that audiovisual elements can mitigate urban stressors, such as highly artificial environments, thereby promoting cognitive restoration and psychological well-being (Zhu et al., 2023). For example, a carefully designed under-bridge space with pleasant landscapes and comfortable seating can enhance users’ sense of belonging, relaxation, and well-being, making them more willing to stay and interact. In specific functional areas, such as art exhibition zones or community activity centers, aesthetic quality and the comfort experience it generates directly affect users’ mental pleasure and willingness to participate. Therefore, this study proposes Hypothesis 2.

*H2*: Aesthetics and comfort in under-bridge spaces have a significant positive effect on users’ mental perception.

Reachability and catalysis (RC) emphasize the connection between under-bridge spaces and the surrounding urban network. This dimension includes transport convenience, path legibility, and ease of access, as well as the capacity of the space to link with community activities, public transport, information guidance, and other elements. As part of the urban fabric, the connectivity of under-bridge spaces with surrounding areas and their own accessibility are crucial (Frank et al., 2006). Cho (2021) argue that improving accessibility and diversity is a key measure for activating idle spaces under viaducts. Previous studies also show that perceived accessibility and connectivity in public spaces are closely related to users’ mental perception. Wu (2019) regards residual spaces under expressway viaducts as landscape nodes capable of catalyzing area development, and advocates integrated spatial transformation across four dimensions: coordination, ecology, functional space, and culture. When under-bridge spaces effectively connect different urban areas and facilitate passage, staying, and communication, users are more likely to develop positive feelings of convenience, openness, and integration. Therefore, reachability and catalysis are essential for establishing and maintaining users’ mental perception of under-bridge spaces. This study proposes Hypothesis 3.

*H3*: Reachability and catalysis have a significant positive effect on users’ mental perception.

### 2.3 Relationships between physical environmental elements and everyday behavioral reshaping

Function and safety (FS) are the fundamental prerequisites for stimulating positive behavior in under-bridge spaces (Felix and Organista, 2024). According to environment-behavior theory, the implantation of spatial functions directly determines what people can do, while a sense of safety determines whether they dare and are willing to do it. In the past, under-bridge spaces often lacked clear functions and had public safety risks, which mainly induced avoidance, escape, and other evasive behaviors. In light of Habermas’s arguments on communicative rationality and consensus building (McFarlane, 2010), creating physical settings that support productive interaction and dialogue can facilitate communication, rest, recreation, and other behaviors.

The literature indicates that when under-bridge spaces are endowed with compound practical functions such as sports fields, children’s play areas, and community convenience facilities, they can directly attract target groups with specific needs, thereby transforming previously idle spaces into frequently used activity places. Studies also show that the improvement of safety elements, including all-weather lighting systems, open sightline corridors, and physical separation from vehicular disturbance, can effectively reduce people’s fear of darkness and the unknown (Barlow et al., 2021). When functions are enriched and safety is secured, behavioral patterns shift from simple passage to purposeful participation and longer stays. This study proposes Hypothesis 4.

*H4*: Function and safety in under-bridge spaces have a significant positive effect on users’ behavioral reshaping.

Aesthetics and comfort (AC) play a key role in transforming necessary behaviors such as passage into spontaneous behaviors such as social interaction. From the perspective of encouraging social activities, Mehta (2007) proposes that street environments should provide a sense of safety, practicality and convenience, comfort in both natural and built environments, pleasure, and belonging. Even when an under-bridge space has basic functions and safety, a lack of beauty and comfort makes it difficult to generate sustained spatial attachment. Numerous studies on urban micro-renewal show that the environmental aesthetic quality of artistic graffiti, pavement texture, and distinctive landscapes can trigger beauty-oriented behavior and attract pedestrians to stop, take photographs, and observe (Luo and Wang, 2021). Meanwhile, suitable sound and light microclimates and ergonomically designed seating directly determine the length of stay. Visual and auditory stimuli can significantly influence people’s emotions (Yin et al., 2023). When an under-bridge space becomes a place with visual appeal and bodily protection, users’ behavioral characteristics are significantly reshaped, further giving rise to more complex forms of social interaction such as conversation and watching others’ activities. This study proposes Hypothesis 5.

*H5*: Aesthetics and comfort in under-bridge spaces have a significant positive effect on users’ behavioral reshaping.

Reachability and catalysis (RC) constitute the physical network basis for breaking spatial isolation and realizing behavioral reshaping (Borst et al., 2008). Gehl (2002) argues in Life Between Buildings that improving the accessibility and comfort of street spaces can promote the diversity of social interaction and behavioral activities. Service facilities, parks, gardens, and other built-environment elements in public spaces can, to some extent, encourage walking and other social activities (Chudyk et al., 2017). In The Death and Life of Great American Cities, Jane Jacobs emphasized the importance of “eyes on the street” in fostering social interaction and a sense of safety, arguing that vibrant streets and diverse public activities can significantly enhance people’s feelings of safety and belonging (Sheng et al., 2018). For under-bridge spaces, transport reachability means safe access through slow-traffic systems and barrier-free routes, while catalysis means convenient connection with surrounding communities and public transport stops. Research suggests that when reachability and catalysis are improved, under-bridge spaces gradually transform into social spaces for commuting, resting, strolling, and interaction. This increase in accessibility not only greatly increases pedestrian flow, but more importantly changes users’ movement trajectories and spatial-use habits, making chance encounters and spontaneous gatherings possible. This study proposes Hypothesis 6.

*H6*: Reachability and catalysis in under-bridge spaces have a significant positive effect on users’ behavioral reshaping.

### 2.4 Mediating role of mental perception

The theoretical framework proposed by Lattman et al. (2016) emphasizes the link between perceptual dimensions and behavioral patterns. In this study, users’ psychological perceptions refer to their subjective experiences of safety, comfort, identity, belonging, and sense of place within under-bridge spaces (Alfonzo, 2005). These psychological perceptions stem from the extent to which individuals perceive the environment as having restorative potential (Kaplan and Kaplan, 1989), which in turn influences whether they are willing to enter, stay, interact, and revisit the space. Kaplan (1995) explained the restorative characteristics of environments from the perspectives of being away, fascination, extent, and compatibility (Herzog et al., 2003). From the perspective of environmental psychology, Haslam argues that crowd perception influences community psychological well-being by fostering social engagement and promoting cognitive restoration (Haslam et al., 2024). From an emotional standpoint, emotional restoration in crowds is primarily manifested through cognitive activities and socialization; a high-quality built environment can alleviate urban stress and facilitate social interaction by cultivating positive emotions (Bornioli et al., 2025). The psychological perception of the crowd directly influences their behavioral reshaping within under-bridge spaces. Indeed, this perception serves as the foundation for assessing whether an under-bridge space is worth revisiting, lingering in, and engaging in activities. Higher levels of psychological perception increase the likelihood of individuals re-entering, staying for extended periods, and actively participating. Particularly against the backdrop of the increasing emphasis on urban public spaces, this perception emerges as a crucial predictor of crowd behavioral reshaping. Therefore, users’ mental perception has a direct effect on behavioral reshaping in under-bridge spaces. This study proposes Hypothesis 7.

*H7*: Users’ mental perception in under-bridge spaces directly affects their behavioral reshaping.

In the model of this study, physical environmental elements may influence user behavior not only directly, but also indirectly through the psychological pathway of mental perception. First, function and safety provide users with basic action conditions and risk control, helping them form a sense of reassurance, trust, and spatial reliability. Such positive mental perception further increases their length of stay, activity participation, and willingness to reuse the space (Yang and Tang, 2025). Therefore, physical environmental elements may directly promote behavioral reshaping and may also indirectly influence behavioral reshaping by enhancing mental perception. This study proposes Hypothesis 8a.

*H8a*: Users’ mental perception mediates the relationship between function and safety and behavioral reshaping in under-bridge spaces.

Second, users’ mental perception mediates the relationship between aesthetics and comfort and behavioral reshaping in under-bridge spaces. Some scholars argue that good environmental aesthetics can stimulate positive emotional experience and place identity, thereby promoting more active spatial-use behavior (Wu et al., 2023). Similarly, improving the aesthetics and comfort of under-bridge spaces helps enhance users’ positive mental perception of the space, such as pleasure, relaxation, and identification, thereby strengthening behavioral reshaping (Huang et al., 2025; Zhang et al., 2026). In the context of under-bridge spaces, the mediating role of users’ mental perception is crucial in the relationship between aesthetics and comfort and behavioral reshaping. This study proposes Hypothesis 8b.

*H8b*: Users’ mental perception mediates the relationship between aesthetics and comfort and behavioral reshaping in under-bridge spaces.

Finally, reachability and catalysis not only alter the physical connection between under-bridge spaces and surrounding urban networks, but also affect users’ judgments of spatial openness, convenience (Chen et al., 2016), and integration. When an under-bridge space has legible paths, convenient entrances, and effective connections with surrounding communities, users are more likely to develop psychological expectations that they are willing to enter and able to participate (Ashihara, 2006), which further increases the likelihood of staying, participating in activities, and socializing. Therefore, users’ mental perception may mediate the relationship between reachability and catalysis and behavioral reshaping in under-bridge spaces. This study proposes Hypothesis 8c.

*H8c*: Users’ mental perception mediates the relationship between reachability and catalysis and behavioral reshaping in under-bridge spaces.

**Figure 2:**
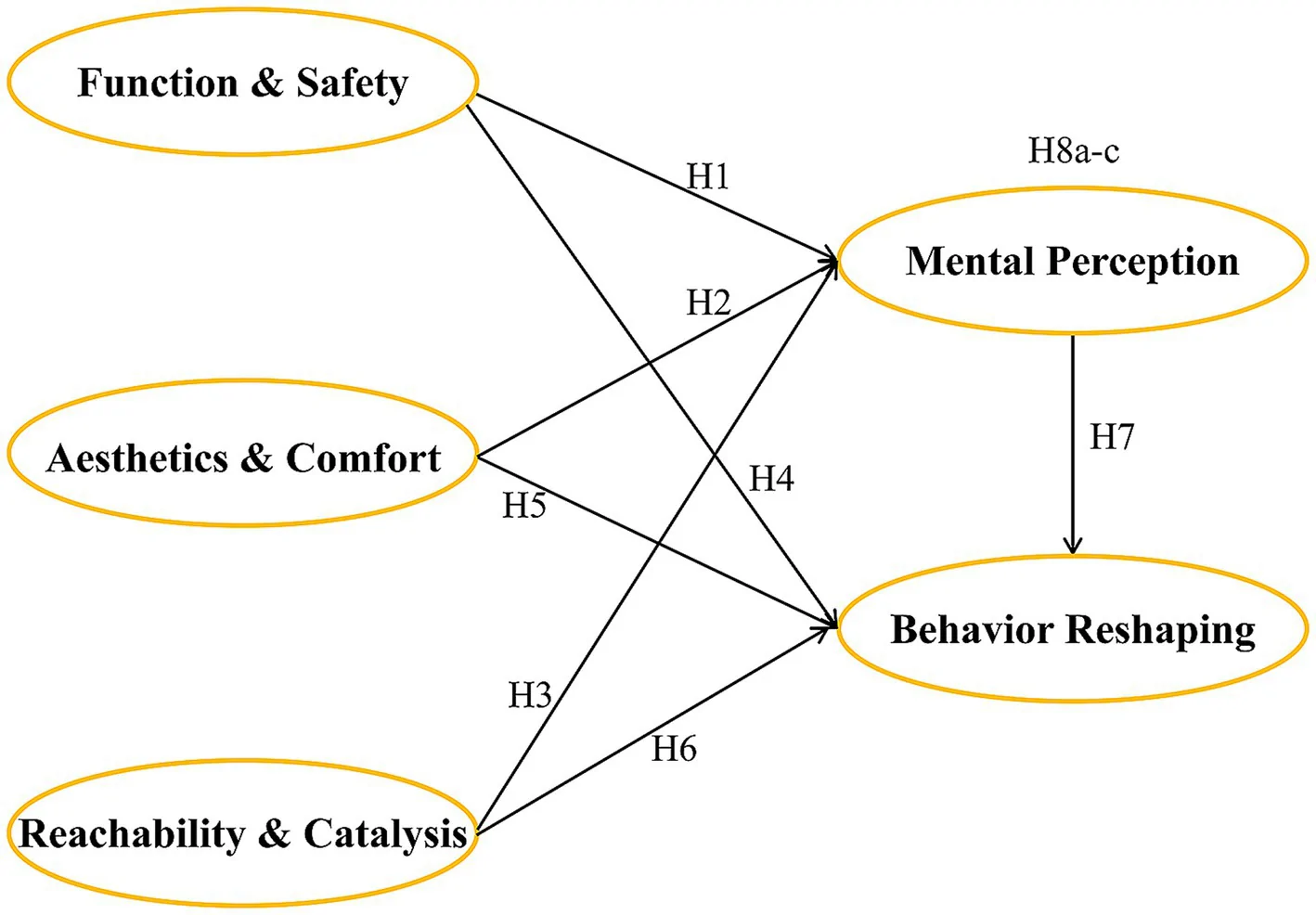
Presents the full research framework and hypotheses of this study (Source: Authors’ own work).

As shown in Figure 2, all hypotheses proposed in this study are presented.

## 3 Research methods

### 3.1 Research design

**Figure 3:**
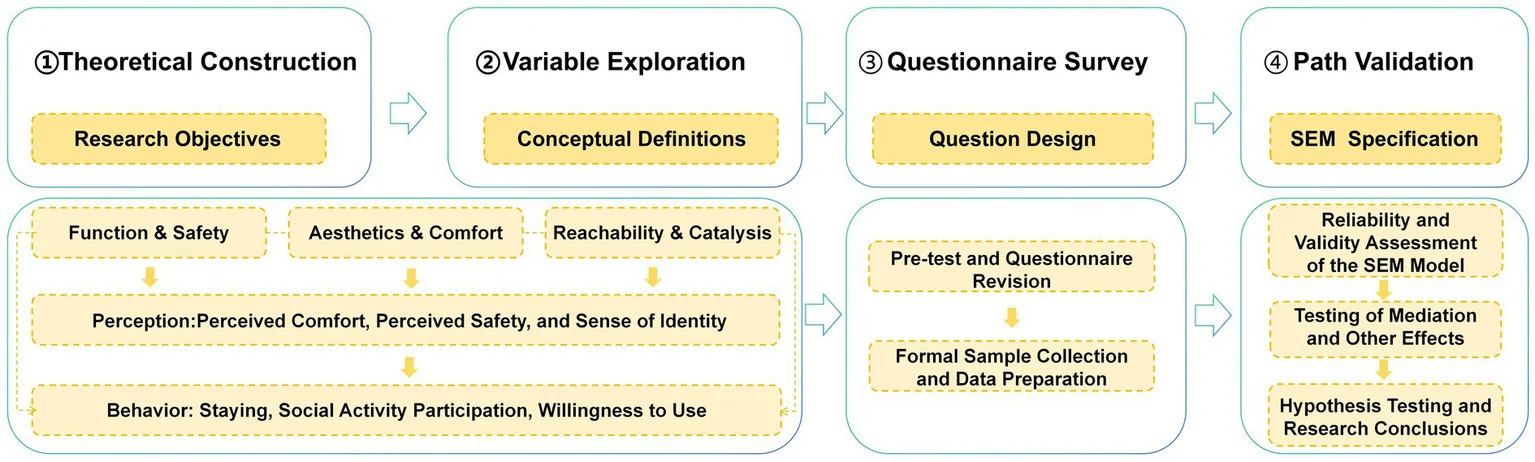
Research design pathway diagram (Source: Authors’ own work).

This study adopts Environment-Behavior Theory as its theoretical framework, employing SEM to investigate the action pathways of “Physical Environment — Psychological Perception — Behavioral Reshaping” within urban lost spaces beneath bridges. The research follows a systematic logic encompassing theoretical construction, variable exploration, questionnaire survey, and pathway validation (Figure 3).

### 3.2 Study subjects and data sources

**Figure 4:**
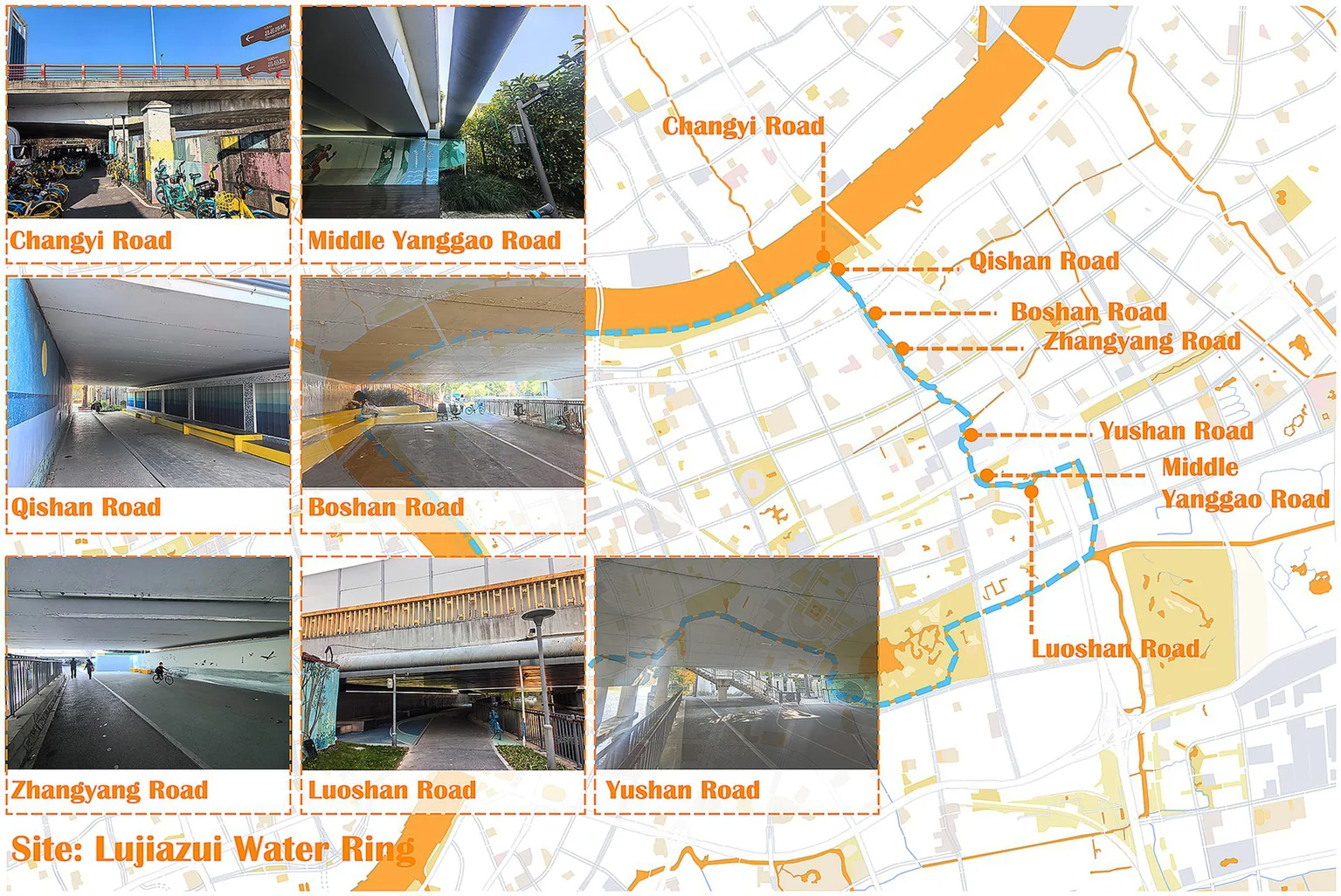
Distribution of research area and psychological perception & behavioral observation points in the Lujiazui Water Ring (Source: Authors’ own work).

This study selected the under-bridge spaces of the Lujiazui Water Ring in Shanghai and the surrounding residents and workers as the survey population. The area is approximately 12 km long and covers seven sites, including Changyi Road, Qishan Road, Boshan Road, Zhangyang Road, Yushan Road, Middle Yanggao Road, and the Luoshan Road section of the Inner Ring Elevated Road (Figure 4). Moreover, within this space, lighting improvements, landscape enhancements, and the addition of seating and recreational facilities—alongside transportation infrastructure, pedestrian circulation, public activities, and residual spaces—converge and overlap here. It therefore covers groups with diverse cultural backgrounds, including individuals with low, middle, and high socioeconomic status. To ensure that the sample was representative of the actual user population’s characteristics (Aithal and Aithal, 2020), the research team conducted intercept questionnaire surveys with space users at various time intervals on both weekdays and weekends. The target population consisted of individuals who entered, passed through, or stayed within the study site during the survey period, including local residents, office workers, commuters, tourists, and other temporary users. Respondents were included if they met the following criteria: (a) being at least 18 years old, which reflects their capacity to comprehend the questionnaire content.; (b) living or working in the community; and (c) voluntarily participating in the study.

This study collected data by distributing questionnaires on site, a survey method that has been effectively applied in previous studies (Aithal and Aithal, 2020). A total of 396 questionnaires were collected; after invalid responses were excluded, 357 valid questionnaires remained. The study adopted a quantitative research design to explore the relationships among function and safety, aesthetics and comfort, reachability and catalysis, users’ mental perception, and everyday behavioral reshaping in under-bridge spaces. The research framework was constructed on the basis of environment-behavior theory. A structured questionnaire was used, and its sections corresponded to the key concepts in the research model. Function and safety, aesthetics and comfort, and reachability and catalysis were measured using a Likert scale (Sullivan and Artino, 2013), ranging from strongly disagree (1), disagree (2), somewhat agree (3), agree (4), to strongly agree (5). The measurement of users’ psychological perceptions in this study was guided by the conceptual framework of the Perceived Restorativeness Scale (PRS). To minimize respondent fatigue caused by an excessive number of items, an abbreviated version was adopted. This abbreviated approach has been validated by Negrín et al. (2017) as a reliable and sensitive instrument for assessing urban environments and has demonstrated a strong correlation with the original psychometric scale (Hartig et al., 1997). The measurement items used in this study are shown in Table 1.

**Table 1:** Measurement items used in this study.

Construct	Indicator	Source
Function and safety	This under-bridge space provides diverse functions, such as rest, exhibition, and sports.	Lak et al. (2019)
	The supporting facilities of this under-bridge space, such as seating, lighting, and waste bins, are complete.	Wu (2019)
	The design of this under-bridge space ensures safe passage for pedestrians and cyclists.	Cerin et al. (2006)
	The light environment of this under-bridge space, including both daytime and nighttime conditions, is comfortable and pleasant.	Yin (2012)
Aesthetics and comfort	The color composition of this under-bridge space is harmonious and artistically appealing.	Song and Xiao (2025)
	The materials used in this under-bridge space have a good texture and enhance spatial quality.	Chen et al. (2016)
	The greening level of this under-bridge space is good.	Ma et al. (2026)
	The sound environment of this under-bridge space is relatively quiet and effectively isolates external noise.	Attoe and Logan (1989)
Reachability and catalysis	This under-bridge space is easy to enter and pass through, with no obvious barriers.	Van der Vlugt et al. (2019)
	The signage system of this under-bridge space is clear and helps me to navigate.	Zhou et al. (2024)
	The design of this under-bridge space is attractive and can help activate the space.	Attoe and Logan (1989)
	The under-bridge space can promote the coordinated development of the surrounding streets and community.	Attoe and Logan (1989)
Mental perception	This place allows me to forget my daily responsibilities, feel relaxed, and become immersed in self-reflection.	Kaplan (1995), Herzog et al. (2003), Hartig et al. (1997), Negrín et al. (2017)
	Fascination: There are many things here worth exploring and discovering.	
	This is a place where activities and things are well organized, making it easy to find your way around.	
	This place feels like another world where I can move around freely.	
Behavioral reshaping	I have seen or participated in informal activities, such as street dance, vending, or performances, in this under-bridge space.	Oldenburg (1999)
	I tend to stay longer in this under-bridge space or choose it as part of my activity route.	Lefebvre (1974)
	A good spatial environment helps stimulate positive public behavior.	Gehl (2002)
	I am willing to participate in the maintenance, management, or public-affairs discussion of this under-bridge space.	Lefebvre (1974)

## 4 Results

### 4.1 Reliability and validity analysis

**Figure 5:**
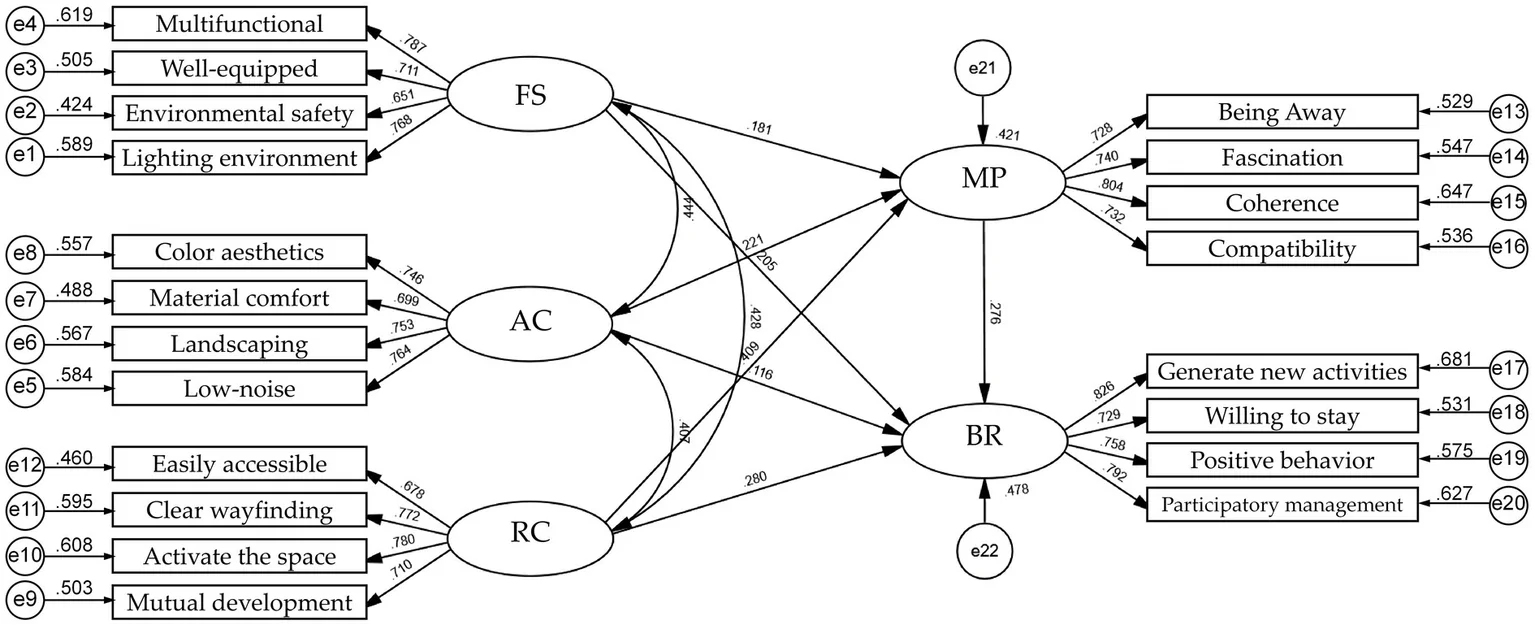
Structural path model (Source: Authors’ own work).

This study used SPSS 26.0 and AMOS 26.0 to conduct empirical tests. Cronbach’s alpha and composite reliability (CR) were used to examine scale reliability, while average variance extracted (AVE) was used to assess convergent validity. As shown in Table 2, the Cronbach’s alpha coefficients of all dimensions were greater than 0.8, and the overall reliability was 0.904, indicating good reliability among the questionnaire items (Hair et al., 2022). For validity testing, KMO and Bartlett indicators were first selected to examine the suitability of the sample data for factor analysis. As shown in the table, the KMO values of all dimensions were greater than 0.7, and the overall validity was 0.897, confirming that the questionnaire results were suitable for validity analysis and for subsequent calculation of factor loadings on the latent variables (Chung et al., 2004). The final test results show that the standardized loadings of all items were greater than 0.6; the CR values of the latent variables ranged from 0.821 to 0.859, exceeding the standard of 0.7; and the AVE values ranged from 0.535 to 0.604, exceeding the standard of 0.5. These results indicate that the scale has high reliability and validity (Hair et al., 2021). By evaluating path coefficients and significance levels, this study comprehensively reveals the relationships among variables and the overall model fit in research on under-bridge spaces. Figure 5 presents the structural path model used in this study.

**Table 2:** Convergent validity and construct reliability.

Variable	Measurement dimension	Factor loading	Cronbach’α	KMO	CR>0.7	AVE>0.5
Function and safety	FS1	0.787	0.818	0.800	0.821	0.535
	FS2	0.711				
	FS3	0.651				
	FS4	0.768				
Aesthetics and comfort	AC1	0.746	0.829	0.804	0.833	0.556
	AC2	0.699				
	AC3	0.753				
	AC4	0.764				
Reachability and catalysis	RC1	0.678	0.823	0.805	0.825	0.542
	RC2	0.772				
	RC3	0.780				
	RC4	0.710				
Mental perception	MP1	0.728	0.838	0.817	0.838	0.565
	MP2	0.740				
	MP3	0.804				
	MP4	0.732				
Behavioral reshaping	BR1	0.826	0.858	0.811	0.859	0.604
	BR2	0.729				
	BR3	0.758				
	BR4	0.792				

As shown in Table 3, discriminant validity was assessed according to the Fornell and Larcker (1981a) criterion. The square root of the AVE value for each variable was greater than the correlation coefficients between that variable and other variables, indicating that the questionnaire scale had good discriminant validity (Henseler et al., 2015).

**Table 3:** Discriminant validity.

Construct	RC	AC	FS	MP	BR
RC	0.736				
AC	0.407	0.746			
FS	0.428	0.444	0.731		
MP	0.576	0.468	0.454	0.752	
BR	0.574	0.450	0.502	0.585	0.777

### 4.2 Overall model fit analysis

Model fit refers to the degree of consistency between the hypothesized theoretical model and the actual data. It is used to evaluate the agreement between model-predicted results and observed data in structural equation modeling (Fornell and Larcker, 1981b). This study selected several commonly used indicators, including the chi-square/degrees of freedom ratio, RMSEA, GFI, NFI, RFI, and CFI, to test model fit. As shown in Table 4, all indicators met the recommended standards.

**Table 4:** Model fit test.

Fit index	χ^2^/df	RMSEA (<0.08)	GFI (>0.9)	NFI (>0.9)	RFI (>0.9)	CFI (>0.9)
Result	1.237	0.026	0.950	0.939	0.928	0.988

### 4.3 Model path coefficients

Model path coefficients indicate the strength of relationships between variables. The magnitude of a path coefficient reflects the strength of the relationship between variables; values usually range from −1 to 1, with positive values indicating positive correlations and negative values indicating negative correlations.

As shown in Table 5, the results supported six hypotheses (MP ← FS, MP ← AC, MP ← RC, BR ← FS, BR ← RC, and BR ← MP), while one hypothesis (BR ← AC) was not supported. The structural equation modeling results show that FS, AC, and RC significantly influenced the mental perception (MP) of under-bridge space users, and that FS, RC, and users’ mental perception jointly had significant effects on user behavior.

**Table 5:** Model path coefficient test results.

Path	Unstandardized estimate	Standardized estimate	*p* values	Result
MP ← FS	0.178	0.181	0.006	Supported
MP ← AC	0.215	0.221	***	Supported
MP ← RC	0.406	0.409	***	Supported
BR ← FS	0.245	0.205	0.001	Supported
BR ← AC	0.137	0.116	0.063	Not Supported
BR ← RC	0.338	0.280	***	Supported
BR ← MP	0.336	0.276	***	Supported

*** indicates that the *p*-value for this path is less than 0.001 (*p* < 0.001).

According to the hypotheses and structural equation model discussed above, this study argues that function and safety (FS), aesthetics and comfort (AC), and reachability and catalysis (RC) not only directly affect behavioral reshaping among under-bridge space users, but also influence behavioral reshaping through users’ mental perception. It is therefore necessary to test the mediating effect of mental perception. This study used the bootstrap method to test the mediating effect of mental perception, with 5,000 resamples and a 95% confidence level for both bias-corrected and percentile confidence intervals. A confidence interval that does not include 0 indicates that the corresponding indirect effect exists (Hair et al., 2022). The test results are shown in Table 6.

**Table 6:** Mediation effect test results.

Path	Bias-corrected 95%	Percentile 95%			Result
	Lower	Upper	Lower	Upper	
FS → MP → BR	0.016	0.138	0.010	0.124	Supported
FS→ BR	0.088	0.409	0.018	0.141	Supported
Total effect (Direct / Indirect)	0.148	0.463	0.051	0.233	Supported
AC → MP → BR	0.024	0.151	0.090	0.410	Supported
AC→ BR	−0.010	0.288	−0.007	0.290	Not Supported
Total effect (Direct / Indirect)	0.067	0.361	0.162	0.531	Supported
RC → MP → BR	0.056	0.240	0.149	0.464	Supported
RC → BR	0.160	0.528	0.066	0.360	Supported
Total effect (Direct / Indirect)	0.307	0.639	0.312	0.644	Supported

For function and safety (FS), mental perception (MP), and behavioral reshaping (BR), the bias-corrected 95% confidence intervals for the direct effect [0.088, 0.409] and indirect effect [0.016, 0.138] did not include 0. The percentile 95% confidence intervals for the direct effect [0.018, 0.141] and indirect effect [0.010, 0.124] likewise did not include 0, indicating the existence of a mediation effect.

Because both the direct and indirect effects were significant, MP played a partial mediating role in the effect of FS on BR.

For aesthetics and comfort (AC), mental perception (MP), and behavioral reshaping (BR), the bias-corrected and percentile 95% confidence intervals for the direct effect were [−0.010, 0.288] and [−0.007, 0.290], respectively, both including 0. This indicates that, after the mediating variable MP was introduced, the direct effect of AC on BR was no longer significant. However, the indirect effect intervals [0.024, 0.151] and [0.090, 0.410] did not include 0, demonstrating that MP had a significant mediating effect in the relationship between AC and BR.

Because the indirect effect was significant while the direct effect was not, users’ mental perception (MP) played a full mediating role in the effect of aesthetics and comfort (AC) on behavioral reshaping (BR). This means that the effect of AC on BR was realized almost entirely through users’ mental perception.

For reachability and catalysis (RC), mental perception (MP), and behavioral reshaping (BR), the bias-corrected 95% confidence intervals for the direct effect [0.160, 0.528] and indirect effect [0.056, 0.240] did not include 0. The percentile 95% confidence intervals for the direct effect [0.066, 0.360] and indirect effect [0.149, 0.464] also did not include 0, indicating the existence of a mediation effect.

Similar to FS, both the direct and indirect effects were significant, indicating that MP played a partial mediating role in the effect of RC on BR.

## 5 Discussion

Using SEM analysis, this study examined the relationships among physical environmental elements, mental perception, and behavioral reshaping in under-bridge spaces. Overall, the findings verified most of the research hypotheses and clarified the key finding that the direct path from aesthetics and comfort to behavioral reshaping was not supported. Specifically, function and safety (FS), aesthetics and comfort (AC), and reachability and catalysis (RC) all significantly enhanced users’ mental perception (MP). Function and safety (FS) and reachability and catalysis (RC) directly influenced behavioral reshaping (BR) and also exerted partial mediating effects through mental perception. Although aesthetics and comfort (AC) did not directly drive behavioral reshaping, they exerted an indirect effect through mental perception. This suggests that the behavioral effects of under-bridge space renewal do not arise solely from the physical environment itself, but depend to a substantial extent on users’ psychological translation of spatial meaning, safety, comfort, and belonging.

### 5.1 Function and safety: physical spatial optimization promotes behavioral participation

Reasonable design of movement routes, activity venues, lighting facilities, and safety provisions in under-bridge spaces can significantly influence users’ mental perception and behavioral performance. The results show that function and safety have a significant direct effect on behavioral reshaping. When the functional layout of an under-bridge space is reasonable, facilities are well configured, and spatial safety is ensured, the space itself provides the necessary conditions for staying, interaction, and activity participation, thereby promoting positive behavior.

At the same time, mental perception played a partial mediating role in this process. This indicates that high-quality spatial function not only provides practical convenience at the physical level, but also indirectly strengthens users’ behavioral investment by shaping positive psychological experiences, such as perceived spatial vitality, safety protection, and belonging. In other words, functional design operates through a dual transmission pathway: it directly enables behavior through the physical space and indirectly stimulates deeper participation through psychological empowerment.

Therefore, the design of under-bridge spaces should not treat functional provision as a simple accumulation of facilities. Instead, it should integrate functions into the overall organization of users’ behavioral paths and psychological experience. While meeting basic functional needs, designers should use clear movement routes, stable lighting, explicit activity boundaries, and continuous spatial sequences to allow users to experience convenience together with vitality, safety, and belonging, thereby achieving a transformation from functional satisfaction to behavioral activation.

### 5.2 Aesthetics and Comfort: behavioral transformation mediated by mental perception

Aesthetics and comfort, which include environmental cleanliness, greening landscapes, art installations, lighting design, and the comfort of resting facilities, constitute a key dimension shaping the use experience of under-bridge spaces. The study found that the direct effect of aesthetics and comfort on behavioral reshaping was not significant; its effect path was instead characterized by full mediation through mental perception.

This finding reveals an important design logic. In the sample of this study, the comfort and aesthetic quality of under-bridge spaces did not directly drive users’ behavioral performance. They first had to be translated into positive mental experience. Specifically, only when environmental comfort and aesthetic features evoke pleasure, relaxation, inspiration, or emotional resonance can the enhancement of mental perception further promote staying, social interaction, and even creative behavior.

Therefore, aesthetic construction and comfort improvement in under-bridge spaces should not remain at the surface level of physical optimization, such as environmental tidiness, facility improvement, and landscape beautification. Greater attention should be paid to users’ psychological feelings and emotional experiences in the space. Design should use pleasant greening landscapes, recognizable art installations, and comfortable, friendly resting facilities to enhance users’ pleasure, belonging, and emotional connection. Only when aesthetics and comfort are transformed into a perceptible, memorable, and identifiable place experience are they more likely to stimulate willingness to stay, communicate, interact, and participate.

### 5.3 Reachability and catalysis: dual drivers of physical convenience and psychological openness

The results show that reachability and catalysis, covering transport convenience, community integration, and the clarity of internal circulation in under-bridge spaces, are core elements for attracting pedestrian flows and promoting effective spatial use. Reachability and catalysis had a significant direct effect on behavioral reshaping, indicating that under-bridge spaces are more likely to transform from marginal spaces that people passively pass through into public spaces that people actively enter and continuously use only when they are embedded in surrounding communities, streets, and public transport networks.

It is noteworthy that mental perception played a partial mediating role in this process. This means that high-quality accessibility and connectivity provide not only physical convenience, but also indirectly strengthen users’ behavioral performance by shaping positive psychological experiences, such as a sense of openness and inclusiveness, ease of integration, and facilitation of social connection.

Therefore, under-bridge space design should advance physical connection and psychological openness simultaneously. On the one hand, traffic organization should be improved, walking routes optimized, entrance recognition strengthened, and connection efficiency with surrounding communities enhanced to improve accessibility and convenience of use. On the other hand, clear wayfinding, open interfaces, friendly scales, and diverse activity nodes should be used to create psychological convenience, openness, and intimacy, making the process of entering and using the space natural, smooth, and pleasant, and thereby stimulating diverse behaviors such as staying, communication, interaction, and participation.

## 6 Conclusion

Focusing on the process through which lost under-bridge spaces move from physical repair to mental resonance, this study clarified the pathway by which function and safety (FS), aesthetics and comfort (AC), and reachability and catalysis (RC) influence users’ behavioral reshaping, and highlighted the mediating role of mental perception (MP). The findings show that different spatial elements influence user behavior through different pathways. Function and safety, as well as reachability and catalysis, can directly affect behavioral reshaping and can also indirectly strengthen behavioral transformation by enhancing users’ psychological experience and spatial identification. Aesthetics and comfort, by contrast, cannot be directly translated into behavioral reshaping; rather, they operate through mental perception, including pleasure, relaxation, belonging, and emotional connection. This indicates that under-bridge space design is not merely a matter of physical environmental transformation. It is a complex process in which spatial form, functional configuration, safety assurance, aesthetic experience, and modes of connection jointly act on the individual’s mental level and ultimately trigger behavioral change. That is from Perception to Practice: Evidence-based Design Guidelines, an associative pathway through which the physical environment evokes users’ mental perceptions, which in turn influence behavioral reshaping.

In summary, the theoretical contribution of this study lies in shifting the renewal of under-bridge spaces from a purely physical transformation problem to a pathway analysis of environmental perception, psychological translation, and behavioral reshaping, further demonstrating the bridging role of mental perception in the renewal of urban under-bridge spaces. In practical terms, the study suggests that future under-bridge space design should adhere to a human-centered approach. On the basis of improving functional diversity, safety assurance, transport connection, and environmental beautification, it should place greater emphasis on users’ psychological experience, emotional needs, and social interaction. Only by creating safe, friendly, open, inclusive, comfortable, aesthetically pleasing, and participatory spatial environments can under-bridge spaces be transformed from inefficient and negative transport appendages into urban public spaces with lasting appeal. This study also has certain limitations. First, the sample was mainly drawn from specific under-bridge spaces in Shanghai and their surrounding populations; therefore, the generalizability of the findings requires further testing. The timeliness, comprehensiveness, and robustness of some scales should also be strengthened, and future studies should continue to refine the relevant scales. Second, the mediating variables included in this study are not exhaustive. Future research may introduce additional latent variables and combine longitudinal data or multi-case comparisons to further test the dynamic relationship between spatial renewal and behavioral change.

## Data Availability

The raw data supporting the conclusions of this article will be made available by the authors, without undue reservation.
